# Access to family planning services and associated factors among young people in Lira city northern Uganda

**DOI:** 10.1186/s12889-024-18605-8

**Published:** 2024-04-24

**Authors:** Eustes Kigongo, Raymond Tumwesigye, Maxson Kenneth Anyolitho, Marvin Musinguzi, Gad Kwizera, Everlyne Achan, Caroline Kambugu Nabasirye, Samson Udho, Amir Kabunga, Bernard Omech

**Affiliations:** 1 Faculty of Public Health, Lira University, Lira City, Northern P. O Box 1035, Uganda; 2 Faculty of Nursing , Mbarara University of Science and Technology, Mbarara, Uganda; 3 Faculty of Education, Lira University, Lira, Uganda; 4 Faculty of Medicine, Lira University, Lira, Uganda

**Keywords:** Access, Contraception, Family planning, Youths, Young people

## Abstract

**Background:**

Access to family planning services among young people is crucial for reproductive health. This study explores the access and associated factors among young people in Lira City, Northern Uganda.

**Methods and materials:**

A mixed-methods study was conducted in March to April 2022. Quantitative data were collected using a structured questionnaire from 553 participants aged 15–24 years. Qualitative data were obtained through in-depth interviews and focus group discussions. Data analysis included univariate, bivariate, and multivariate analyses for quantitative data, while interpretative phenomenological analysis was used for qualitative data.

**Results:**

Overall, 31.7% of the respondents had a good perceived access to family planning services, with 64.6% reporting perceived availability of FP methods. Challenges included lack of privacy (57.7%), fear of mistreatment (77.2%), and decision-making difficulties (66.2%). Among females, good perceived access to FP services was less likely among urban residents (AOR: 0.22, 95% CI: 0.09–0.53), Christian respondents (AOR: 0.51, 95% CI: 0.01–0.36), Muslim respondents (AOR: 0.07, 95% CI: 0.01–0.55) and respondents with poor attitude to FP services (AOR: 0.39, 95% CI: 0.24–0.64), but more likely among respondents with a sexual a partner (AOR: 4.48, 95% CI: 2.60–7.75). Among males, good perceived access to FP services was less likely among respondents living with parents (AOR: 0.19, 95% CI: 0.05–0.67) but more likely among respondents with good knowledge of FP services (AOR: 2.28, 95% CI: 1.02–5.32). Qualitative findings showed that three themes emerged; knowledge of family planning methods, beliefs about youth contraception and, friendliness of family planning services.

**Conclusion:**

The study revealed a substantial gap in perceived access to family planning services among young people in Lira City. Barriers include privacy concerns, fear of mistreatment, and decision-making difficulties. Tailored interventions addressing urban access, religious beliefs for females, and knowledge enhancement for males are essential. Positive aspects like diverse FP methods and physical accessibility provide a foundation for targeted interventions. Youth-friendly services, comprehensive sexual education, and further research are emphasized for a nuanced understanding and effective interventions in Northern Uganda.

**Supplementary Information:**

The online version contains supplementary material available at 10.1186/s12889-024-18605-8.

## Background

Globally, approximately 16 million girls aged 15–19 give birth each year, with 95% of these births occurring in developing countries [1]. Additionally, annually, 14 million unsafe abortions take place among adolescents, who face various sexual and reproductive health challenges, including early pregnancy, unsafe abortions, sexually transmitted infections (STIs), and sexual abuse, particularly in Sub-Saharan Africa (SSA) [2]. Family planning (FP) is a critical aspect of global public health, recognized for its impact on maternal and child health, gender equality, and socioeconomic development [3]. The international community, as reflected in various global health initiatives and sustainable development goals, acknowledges the importance of ensuring universal access to FP services for all individuals, including young people [4]. This global perspective emphasizes the interconnectedness of reproductive health and broader efforts to achieve sustainable development [4]. Access to these services is particularly pertinent among young people, who constitute a significant demographic in many Low-and Middle-income Countries (LMICs).

Uganda, boasting one of the world’s youngest and fastest-growing populations, has nearly half (48%) of its estimated 46 million people under the age of 15, significantly surpassing the averages for SSA (43%) and the world (26%) [5]. Uganda, as a signatory to global health agendas, has made significant strides in promoting FP services [6]. The National Population Policy, coupled with the National Reproductive Health Policy, reflects the government’s commitment to ensuring access to FP for all citizens [7]. However, challenges persist, especially in urban areas. An examination of the national context provides insights into the policy circumstances, healthcare infrastructure, and societal norms that shape family planning services’ availability and utilization among young people in Northern Uganda.

According to the Uganda Demographic and Health Survey (UDHS) 2016, 25% of women aged 15–19 and 1% below 15 had initiated childbearing, with the incidence of unplanned pregnancies significantly rising following the shutdown of schools during the COVID-19 pandemic [8, 9]. Reported underlying causes of teenage pregnancy include gender inequality, restricted freedom for girls to voice their concerns, school dropout, and limited access to contraception and knowledge [10]. Unintended teenage pregnancies can have severe adverse effects on well-being, leading to maternal morbidity and mortality related to childbirth and unsafe abortion [11]. Moreover, these pregnancies contribute to social consequences such as stigma and discrimination, accounting for 59% of school dropouts in Uganda in 2012, potentially hindering education and future employment opportunities [12, 13]. Reports by the World Bank and the World Health Organization (WHO) emphasize the association of adolescents-childbearing with social stigma, lifelong poverty, and health risks, necessitating a comprehensive approach to address these issues [14, 15].

Uganda’s current health sector strategy aims to expand youth-friendly health services (YFHS) and promote adolescent sexual and reproductive health and rights information in schools, ensuring access to FP information and services irrespective of age, marital status, or school status [16]. The country plans to increase access to modern contraceptive use and reduce unmet need for contraception in the coming years [17]. According to WHO guidelines, addressing the underlying factors, including the timing of first sex and marriage, effective contraceptive use, and the socio-cultural and economic environment, is crucial for delaying childbearing and expanding FP access to adolescents [18].

Notably, northern Uganda bears one of the highest burdens of adolescent pregnancies, with reports indicating a significant percentage of unintended pregnancies in Lira district in 2019 (33.3%) and a notable number of teenage girls visiting antenatal clinics in Lango sub-region in 2021 [19]. A recent study in Oyam district reported a high percentage of unintended pregnancies among adolescent girls [8]. In the context of Lira City in northern Uganda, unintended pregnancies represent a significant challenge affecting various aspects of young people’s lives, including education and economic prospects [20]. Despite the recognized importance of FP, there is a need for a comprehensive understanding of the factors hindering or facilitating youth access to FP services in Lira City. Existing literature primarily focuses on the prevalence of unintended pregnancies and associated outcomes, emphasizing challenges in accessing reliable information, contraceptives, and quality reproductive health services [20]. However, there is limited research examining the factors contributing to these challenges, such as cultural norms, stigma, and structural barriers specific to Lira City. Moreover, the evolving circumstances of youth perspectives, preferences, and behaviors related to FP require an updated understanding, considering rapid socio-cultural changes and advancements in technology [21]. To develop evidence-based intervention strategies, our assessment focused on the knowledge, perceptions, and factors influencing access to contraceptive services among young people in the specific context of northern Uganda.

## Methods and materials

### Study design

This was an explanatory-sequential mixed methods study [22] conducted in Lira city, northern Uganda between March and April 2023. The mixed-methods approach was adopted so as to generate a more holistic understanding and a stronger inference with two approaches complementing each other [23].

### Study setting

Lira City is among the newly created cities, located approximately 375 km by road north of the capital city of Kampala via Karuma-Kamdini. Lira City is the central business hub for Northern Uganda and comprises the west and east divisions. According to projections by the Uganda Bureau of Statistics (UBOS) in 2014, the population of 2020 for the Lira district was 474,200 people, and it is traditionally inhabited by the Lango tribe, who are farmers and cattle keepers. The urban centers of the district also have people engaged in many small-scale businesses, such as produce businesses and trading.

### Study population

The study was among young people aged 15 to 24 years, residing in Lira city. Inclusion into the study was based on being a young person of 15 to 24 years of age who has lived in Lira city for at least six months. Additionally, being present at the selected household during data collection, and those who consented to participate were included in the study. In households where more than one persons were eligible, simple random sampling by lottery method was employed to select one. Exclusion was based on being critically ill to participate, or refusing to participate in the interviews.

### Sample size determination

The sample size of the study was estimated using Kish Leslie (1965) as follows:

$$ n=deff*{Z}^{2}*p(1-p)/{d}^{2}$$

In the equation above, n is the sample size for the study, Z is the Z score at the 95% confidence interval (1.96), p is the proportion of perceived access to FP services (50%), d is the desired precision of the study (5%), and deff is the design effect due to multistage random sampling. A factor of 1.5 has been used to adjust the sample size based on what previous studies have used [24, 25]. A design effect of 1.5 was employed to increase the homogeneity of the participants following the use of a multistage random sampling procedure. Therefore, the final sample size obtained was 577.

Interpretative phenomenological analysis (IPA) was employed for qualitative research to delve into individual experiences, progressing towards an examination of shared and contrasting aspects within a limited sample [26]. This approach facilitated the identification of thematic connections. Adhering to IPA guidelines advocating for a compact and homogenous sample, purposive sampling was used to recruit 5 participants. This sample size aligns with the recommended number for an IPA study [27], and was considered sufficient to capture a distinct range of experiences related to the phenomenon under investigation.

### Sampling technique

A multistage sampling procedure was employed to select the 577 study participants. The study was conducted in both divisions of Lira City, East and West. From each of the divisions, five wards were selected, making a total of ten wards. This was done by simple random sampling using the lottery method, where the names of wards were written on small papers, folded, mixed in a container, and shaken well, and then five were picked at random without replacement. From each of the wards, two cells were selected using the same procedure, which generated a total of 20 cells. From each of the cells, Village Health Teams (VHTs) were used to obtain lists of households with young people aged 15 to 24 years, and these were used as sampling frames per cell. The number of participants to be selected from each cell was determined by the sample size proportionate to the cell size. In each of the cells, participants were selected through simple random sampling using computer-generated random numbers. Purposive sampling was used to select participants for qualitative interviews [28]. While purposive sampling guided our selection process, we also sought to include a diverse range of perspectives by engaging with individuals from various backgrounds, including community health workers, educators, and youth leaders. Our rationale for selecting community peer educators stems from their unique position as trusted intermediaries within their communities, often serving as frontline advocates for reproductive health education and services. Similarly, the inclusion of university leaders was motivated by their influence and role in shaping policies and programs related to youth reproductive health within academic settings.

### Study variables

#### Dependent variable

The dependent variable for the study is perceived access to FP services. Access to healthcare means “the timely use of personal health services to achieve the best health outcomes” [29]. Many frameworks have been proposed to measure access to family planning services but have all proved not sufficient [30]. This study adopted one of the common frameworks, Penchansky and Thomas (1981) framework that reflects the fit between characteristics and expectations of the providers and the clients. These characteristics (5As of access) are availability, accessibility, acceptability, accommodation, and affordability [31]. This conceptualization of access has been adopted because it describes the broad dimensions and determinants that integrate demand and supply-side factors [32]. According to the model, the five As of access form a chain that is no stronger than its weakest link. For example, improving affordability by providing health insurance will not significantly improve access and utilization if the other four dimensions have not also been addressed. The perception of access to FP services index composed of five questions of yes or no response. For all the questions “yes” was coded 2 and “no” coded 1. The percentage of respondents that perceived access to be good on all five variables had good perceived access to FP services.

1. Availability: Are the family planning commodities available when you need them, and meet your FP needs?
2. Accessibility: Is the location of the facilities that provide family planning services convenient for you?
3. Acceptability: Are the characteristics of the FP service providers (including attitudes and attributes such as age, sex and religion) comfortable for you?
4. Accommodation: Do health providers organize FP services in ways (including appointment system, hours of operation and facility environment) that suit your needs and preferences?
5. Affordability: Do you have to pay for family planning services?

All the access questions were asked as yes and no questions and coded 1 and 2, respectively. To measure the index of perceived access, only participants who answered Yes to all the access questions were labeled as having good perceived access to FP services.

#### Independent variables

The independent variables included sociodemographic characteristics (age, sex, education, religion, marital status, living with parents), sexual-related characteristics (having a child, sexually active, sexual partners), knowledge, and attitudes. The knowledge of the participants was assessed based on a total of nine questions about family planning. Each of the questions was binary coded as 1(Yes) and 0(No). Overall knowledge was therefore measured as a composite score ranging from 0 to 9. The mean score was taken as a cut-off with individuals above the mean score categorized as having good knowledge and those below the mean as having poor knowledge. This measurement was adopted from a recent study [33]. The overall attitudes of the young people regarding actual use of family planning commodities, which includes the misconceptions, fears, cultural and religious beliefs about family planning commodities such as condoms were assessed based on a total of eight questions with a favorable response coded as Yes (0) and unfavorable response coded as No [1]. The responses were computed into an overall attitude to FP services score with a total of eight. Similarly, the cut-off was set as the mean with individuals above the mean classified as having a poor perception and those below the mean with a good perception, as from a recent related study [33]. The knowledge items had a scale reliability coefficient of 0.78 whereas the perception items had 0.70, all these are within the acceptable limits [34].

### Participant recruitment and informed consent processes

After obtaining ethical approval and clearance, five research assistants from the city were recruited and trained on the study protocol and data collection procedures. A pretest of the questionnaire was carried out among 58 youths from Lira district to refine the questions for simplicity and comprehension and to assess validity and reliability using the Statistical Package for Social Sciences (SPSS) software. Lists of households with young people aged 15 to 24 years were obtained by Village Health Teams (VHTs). Sampling was then conducted, and eligible participants were approached for data collection after providing informed consent and, for minors, informed assent. During this process, the study objectives, procedures, benefits, risks, and voluntarism were explained. Interviews took place in a private space within the participants’ homes. In cases where the parent or guardian was absent during data collection, the household was skipped.

### Data collection instruments

Quantitative data was collected using a pretested interviewer-administered questionnaire developed by the researcher (Supplementary file 1). The questionnaire consisted of four sections: sociodemographic characteristics (age, sex, education, religion, marital status, residence, and parent’s education), sexually related information (ever had a child, engaged in sexual relationships, number of sexual partners, sexual risks encountered), access questions (availability, accessibility, acceptability, accommodation, and affordability), knowledge of family planning services, and attitudes regarding family planning services. This was administered in approximately 15 min. Qualitative data was collected through in-depth interviews and focus group discussions using guides (Supplementary file 2). This was done after obtaining insights from quantitative data. Interviews with participants were done at proposed times and places deemed convenient to the participants themselves. During collection, audio recordings were made together with extended field notes to complement the audios. Data collection was done in Lango, verbatim transcribed, and then translated to English for analysis. Data collection was conducted through five in-depth interviews and four focus group discussions all from young people aged 15 to 24 years. A sample of 10 were from the University and 30 were from the community with equal proportions of males and females. These participants were community adolescent peer-educators and University reproductive health leaders. Some were picked after quantitative interviews while others based on their roles regarding reproductive health for the young people.

### Statistical analysis

#### Quantitative data analysis

The collected data was entered into SPSS software, where it was cleaned and coded, then exported to STATA version 17 software for final analysis. The analysis was conducted at three levels. At the univariate level, data was summarized as frequencies and proportions, means and standard deviations, or median with interquartile range, and presented in frequency tables. In bivariate analysis, perception of access to SRHR services was cross-tabulated with the independent variables one at a time to assess relationships. A crude odd ratio (COR) and a 95% confidence interval were reported. At this level, associations were considered at *p* < 0.25 in order to consider all possible predictors [35], and all those associated factors were taken into multivariate analysis. In multivariate analysis, binary logistic regression was used to estimate the predictors of the primary outcome. The backward elimination method was used to build a predictive model. Results were reported as adjusted odds ratios and 95% confidence intervals. A *p*-value of < 0.05 was considered statistically significant for variables.

#### Qualitative data analysis

The data analysis adhered to the seven-stage IPA process outline, derived from Smith and colleagues, as outlined by Brown and colleagues [36]. Each interview underwent verbatim transcription and was entered into a customized IPA analysis framework. Multiple re-readings of the interviews were conducted, applying in-method triangulation by integrating field notes with observations and commentary from the fieldwork [37]. This triangulation process enhanced confidence in the outcomes post data analysis. Following the verbatim transcription of the audio data and thorough review of the text, initial notes were made, leading to the development of emerging themes. Connections across these emergent themes were sought to identify subordinate themes. Subsequently, a search for patterns across the cases was conducted to reveal the major themes.

### Rigor

We employed research assistants who are social scientists trained in qualitative study and interview techniques to assure the validity of our study. Data from diverse sources, including field notes and audio recordings, were independently analyzed by two researchers. The newly emerging themes were routinely compared to the original transcribed text, and the writers frequently convened for debriefings to make sure that the subjects were at the center of the data analysis and interpretation. The results of the data analysis were examined and discussed until a consensus was achieved in order to increase the dependability and accuracy of the results. To demonstrate confirmability (the degree to which the findings are shaped by participants and the context rather than the perspectives of the research), the researchers used participants’ narratives and words as noted in the transcripts. Additionally, the researchers dwelled on their previous experiences to reduce their influence on the findings. To ensure that the processes of data collecting and analysis could be traced back to the initial interviews, we have preserved all audit trails from data collection to analysis.

## Results

### Quantitative findings

#### Sociodemographic characteristics

Recruitment into the study was between March and April 2022. Out of a total of 577 participants, 553 were included generating a response rate of 95.8%. Table 1 shows that the majority of the respondents, 65.3% were female, with a mean age of 17 (± 2.1) years and 90.8% aged between 15 and 19 years. Most of the youth, 45.2% were in secondary school, 40.7% were Anglican, and 71.4% were living with their parents. The majority of the youths, 46.8% were sexually active and had had sex in the past 4 months.

**Table 1:** Sociodemographic characteristics of respondents

Characteristic	Females *n*(%)	Males *n*(%)	Both *n*(%)
**Age category**			
15–19	330(91.4)	172(89.6)	502(90.8)
20–24	31(8.6)	20(10.4)	51(9.2)
Mean Age (SD)	17.3(± 2.1)	17.5(± 1.9)	17.3(± 2.1)
**Education level**			
None	27(7.5)	23(12.0)	50(9.0)
Primary	136(37.7)	70(36.5)	206(37.3)
Secondary	165(45.7)	85(44.3)	250(45.2)
Tertiary	33(9.1)	14(7.3)	47(8.5)
**Residence**			
Rural	77(21.3)	71(37.0)	148(26.8)
Urban	284(78.7)	121(63.0)	405(73.2)
**Religion**			
Others	27(7.5)	26(13.5)	53(9.6)
Muslim	33(9.1)	30(15.6)	63(11.4)
Christian	301(83.4)	136(70.8)	437(79.0)
**Marital relationship**			
Yes	64(17.7)	17(8.9)	81(14.7)
No	297(82.3)	175(91.2)	472(85.4)
**Live with parents**			
No	104(28.8)	54(28.1)	158(28.6)
Yes	257(71.2)	138(71.9)	395(71.4)
**Mother’s education**			
None	59(16.3)	51(26.6)	110(19.9)
Primary	123(34.1)	84(43.8)	207(37.4)
Secondary	140(38.8)	35(18.2)	175(31.7)
Tertiary	39(10.8)	22(11.5)	61(11.0)
**Father’s education**			
None	34(9.4)	21(10.9)	55(19.0)
Primary	83(23.0)	49(25.5)	132(23.9)
Secondary	134(37.1)	62(32.3)	196(35.4)
Tertiary	110(30.5)	60(31.3)	170(30.7)
**Have a child**			
No	280(77.6)	172(89.6)	452(81.7)
Yes	81(22.4)	20(10.4)	101(18.3)
**Had sex in the past four months**			
No	186(51.5)	108(56.3)	294(53.2)
Yes	175(48.5)	84(43.8)	259(46.8)
**Current number of sexual partners**			
0	206(57.1)	111(57.8)	317(57.3)
1	134(37.1)	40(20.8)	174(31.5)
> 1	21(5.8)	41(21.4)	62(11.2)

### Perceived access to family planning services

**Fig. 1:**
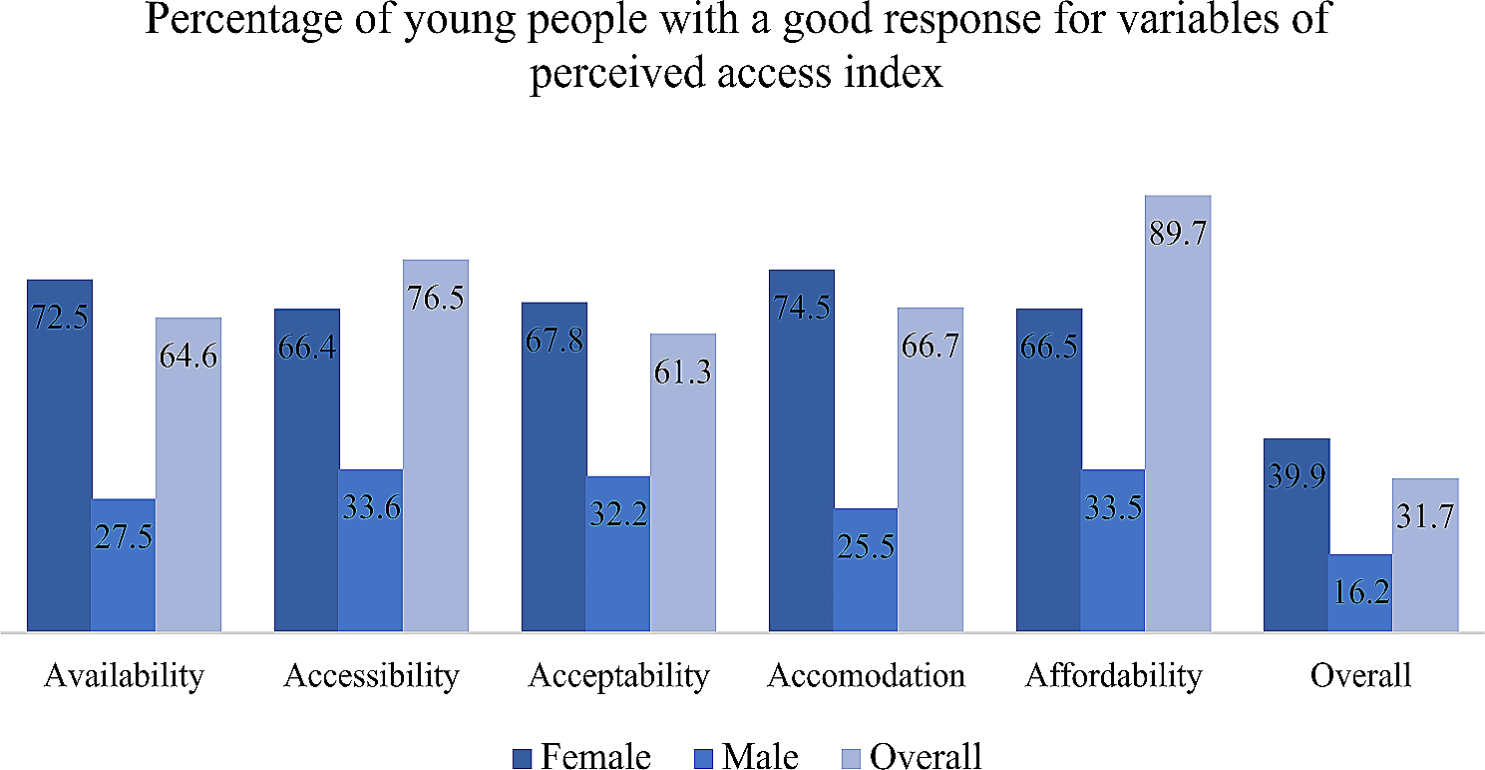
Percentage of young people reporting a good response to variables on the perceived access index in Lira district, Northern Uganda

The mean score for perception of access to family planning services was 1.91 with a standard deviation of ± 0.29. Figure 1 shows that the percentage of respondents that perceived access to be good for all the five variables was 31.7% (95% CI: 28%, 36%). The majority of the young people, 64.6% reported that different FP methods were available at the health facilities. Most of the young people, 79.3%% also reported that the health facilities were within their reach, and 61.3% reported that attitudes and personal characteristics of FP service providers were comfortable for them. The majority of the young people, 66.7% also reported that the manner in which FP services are organized, including facility’s operating and environment, suited their needs and preferences. Additionally, females had overall favorable responses compared to their male counterparts.

### Knowledge and attitudes regarding perceived access to family planning services

Table 2 presents questions used to assess both knowledge and perceptions regarding family planning services. questions 1 to 9 were designed to measure knowledge and 10 to 17 were aimed at capturing perceptions regarding use of family planning commodities. The majority of the young people, 69.4% were aware that FP, 75.6% knew the facility that offers FP services, 89.3% knew how to prevent pregnancy and 75.8% knew about sexual rights. Table 3 also shows that the majority of the young people, 58.1% perceived that FP services were not for young people, 80.5% could access FP whenever they wanted and 90.4% knew that information at the health facility was always kept confidential. However, the majority of the young people, 66.2% cannot decide on using a FP method, 57.7% also reported that there is not enough privacy at the health facilities, and 77.2% fear being mistreated by the staff at the health facilities.

**Table 2:** Knowledge and attitudes of young people regarding perceived access to family planning services in Northern Uganda

Items	Frequency (*n*)	Percentage (%)
Aware of availability of family planning services		
No	169	30.6
Yes	384	69.4
**Knowledge of at least three family planning methods**		
No	12	2.2
Yes	541	97.8
**Knowledge of health facility that offers FP services**		
No	135	24.4
Yes	418	75.6
**Knowledge of health facility that offers abortion services**		
No	342	61.8
Yes	211	38.2
**Knowledge of health facility that offers STIs services**		
No	131	23.7
Yes	422	76.3
**Knowledge of health facility that offers HIV VCT services**		
No	145	26.2
Yes	408	73.8
**Knowledge on how to prevent pregnancy**		
No	59	10.7
Yes	494	89.3
**Knowledge on the rights of young people**		
No	134	24.2
Yes	419	75.8
**Knowledge of the sexual and reproductive rights of young people**		
No	169	30.6
Yes	384	69.4
**Overall knowledge**		
**Poor**	**203**	**36.7**
**Good**	**350**	**63.3**
**FP services are supposed to be used by only married people**		
No	109	19.7
Yes	444	80.3
**Information at the health facility is kept confidential**		
No	53	9.6
Yes	500	90.4
**I can choose my own partners**		
No	13	2.4
Yes	540	97.6
**I can decide on using FP method**		
No	366	66.2
Yes	187	33.8
**I can access FP services whenever I want**		
No	108	19.5
Yes	445	80.5
**There is not enough privacy at the health facility**		
No	234	42.3
Yes	319	57.7
**I fear being embarrassed at the health facility**		
No	329	59.5
Yes	224	40.5
**I fear being mistreated by staff at the health facility**		
No	126	22.8
Yes	427	77.2
**Overall attitude to FP services**		
**Good**	**307**	**55.5**
**Poor**	**246**	**44.5**

### Factors associated with perceived access to family planning services among young people

The bivariate analysis was performed stratified by sex to prevent introduction of bias arising from differencing sample sizes because males were close to a third of the entire sample. Table 3 indicates that among the females, being aged 20–24 years, having a child, being sexually active and having a sexual partner was associated with a higher perceived access to FP services at *p* value less than 0.25. On the other hand, having primary and secondary education, urban residence, Christians, Muslims, not in a marital relationship, secondary education of a mother, primary, secondary and tertiary education of father, and good attitude towards FP services were associated with a lower perceived access to FP services at *p* value less than 0.25. Table 3 also shows that among males, living with parents, mother’s secondary education level, and good attitude towards FP services had a lower perceived access to FP, where as being sexually active and good overall knowledge of FP services had higher perceived access to FP services with *p* value of less than 0.25.

**Table 3:** Factors associated with perceived access to family planning methods among young people in northern Uganda

Characteristic	Female		Crude OR (95% CI)	*P* value	Male		Crude OR (95% CI)	*P* value
	Yes *n*(%)	No *n*(%)			Yes *n*(%)	No *n*(%)		
**Age category**								
15–19	140(97.2)	190(87.6)	1.00		28(90.3)	144(89.4)	1.00	
20–24	4(2.8)	27(12.4)	4.97(1.70-14.54)	0.003*	3(9.7)	17(10.6)	1.10(0.30–4.01)	0.883
**Education level**								
None	0(0)	27(12.4)	1.00		2(6.5)	1(13.0)	1.00	
Primary	65(45.1)	71(32.7)	0.24(0.09–0.63)	0.003*	12(38.7)	58(36.0)	0.46(0.10–2.23)	0.335
Secondary	73(50.7)	92(42.4)	0.28(0.11–0.71)	0.008*	16(51.6)	69(42.9)	0.41(0.09–1.93)	0.260
Tertiary	6(4.2)	27(12.4)	-	-	1(3.2)	13(8.1)	1.24(0.10-15.05)	0.867
**Residence**								
Rural	8(5.6)	69(31.8)	1.00		10(32.3)	61(37.9)	1.00	
Urban	136(94.4)	148(68.2)	0.13(0.06–0.27)	< 0.001*	21(67.7)	100(62.1)	0.78(0.34–1.77)	0.553
**Religion**								
Other	1(0.7)	26(12.0)	1.00		3(9.7)	23(14.3)	1.00	
Christian	133(92.4)	168(77.4)	0.15(0.05–0.42)	< 0.001*	23(74.2)	113(70.2)	0.64(0.18–2.31)	0.497
Muslim	10(6.9)	23(10.6)	0.26(0.08–0.84)	0.025*	5(16.1)	25(15.5)	0.65(0.14–3.04)	0.586
**Marital relationship**								
Yes	11(7.6)	53(24.4)	1.00		2(6.5)	15(9.3)	1.00	
No	133(92.4)	164(75.6)	0.26(0.13–0.51)	< 0.001*	29(93.6)	146(90.7)	0.67(0.15–3.09)	0.609
**Live with parents**								
No	16(11.1)	88(40.6)	1.00		3(9.7)	51(31.7)	1.00	
Yes	128(88.9)	129(59.5)	0.18(0.10–0.33)	< 0.001*	28(90.3)	110(68.3)	0.23(0.07–0.80)	0.020*
**Mother’s education**								
None	13(9.0)	46(21.2)	1.00		5(16.1)	46(28.6)	1.00	
Primary	31(21.5)	92(42.4)	0.84(0.40–1.75)	0.640	13(41.9)	71(44.1)	0.59(0.20–1.78)	0.351
Secondary	87(60.4)	53(24.4)	0.17(0.09–0.35)	< 0.001*	9(29.0)	26(16.2)	0.31(0.10–1.04)	0.057*
Tertiary	13(9.0)	26(13.0)	0.57(0.23–1.40)	0.217	4(12.9)	18(11.2)	0.49(0.18–2.03)	0.325
**Father’s education**								
None	1(0.7)	33(15.2)	1.00		2(6.5)	19(11.8)	1.00	
Primary	24(16.7)	59(27.2)	0.07(0.01–0.58)	0.013*	8(25.8)	41(25.5)	0.54(0.10–2.79)	0.461
Secondary	72(50.0)	62(28.6)	0.03(0.01–0.20)	< 0.001*	11(35.5)	51(31.7)	0.49(0.10–2.41)	0.378
Tertiary	47(32.6)	63(29.0)	0.04(0.01–0.31)	0.002*	10(32.3)	50(31.1)	0.53(0.11–2.63)	0.434
**Have a child**								
No	133(91.0)	149(68.7)	1.00		27(87.1)	145(90.1)	1.00	
Yes	13(9.0)	68(31.3)	4.60(2.43–8.70)	< 0.001*	4(12.9)	16(9.9)	0.74(0.23–2.40)	0.622
**Sexually active**								
No	108(75.0)	78(35.9)	1.00		21(67.7)	87(54.1)	1.00	
Yes	36(25.0)	139(64.1)	5.35(3.35–8.54)	< 0.001*	10(32.3)	74(45.9)	1.79(0.79–4.03)	0.163*
**No. of sexual partners**								
0	118(81.9)	88(40.6)	1.00		23(74.2)	88(54.7)	1.00	
1	25(17.4)	109(50.2)	5.84(3.50–9.78)	< 0.001*	3(9.7)	37(23.0)	3.22(0.91–11.40)	0.069*
> 1	1(0.7)	20(9.2)	26.82(3.53-203.64)	0.001*	5(16.1)	36(22.3)	1.89(0.66–5.33)	0.234*
**Overall knowledge**								
Poor	55(38.2)	74(34.1)	1.00		15(48.4)	59(36.7)	1.00	
Good	89(61.8)	143(65.9)	1.19(0.77–1.85)	0.427	16(51.6)	102(63.4)	1.62(0.74–3.51)	0.221*
**Overall attitude**								
Poor	51(35.4)	144(66.4)	1.00		15(48.4)	97(60.3)	1.00	
Good	93(64.6)	73(33.6)	0.28(0.18–0.43)	< 0.001*	16(51.6)	64(39.7)	0.62(0.29–1.34)	0.223*

OR: odds ratio, CI: confidence interval, *significant *p* value (< 0.25)

Multivariate analysis was performed to assess the predictors of perceived access to family planning services for males and females, presented as two separate models for females and males. Table 4 show among females, residence, religion, sexual partners and perception regarding use of family planning methods had significant associations. Females respondents who were less likely to consider access to family planning services as good were urban residents (AOR: 0.22, 95% CI: 0.09–0.53, *p* = 0.001), those who were Christian (AOR: 0.51, 95% CI: 0.01–0.36, *p* = 0.003) and Muslim (AOR: 0.07, 95% CI: 0.01–0.55, *p* = 0.012), and those who had a poor attitude towards family planning services (AOR: 0.39, 95% CI: 0.24–0.64, *p* < 0.001). Female respondents who had a sexual partner were more likely to consider access to family planning services as good (AOR: 4.48, 95% CI: 2.60–7.75, *p* < 0.001). Table 4 also shows that among males, living with parents and overall knowledge about family planning services were significantly associated with perceive access to family planning services. Male respondents that were less likely to consider access to family planning as good were those who lived with their parents (AOR: 0.19, 95% CI: 0.05–0.67, *p* = 0.010), and those that were more likely to consider access to family planning as good were those that had good overall knowledge about family planning methods (AOR: 2.28, 95% CI: 1.02–5.32, *p* = 0.050).

**Table 4:** Predictors of perceived access to family planning services among young people in Lira city, northern Uganda

Perceived access to FP services	Crude OR(95% CI)	*P* value	Adjusted OR(95% CI)	*P* value
**Model 1– Females**				
**Residence**				
Rural	1.00		1.00	
Urban	0.13(0.06–0.27)	< 0.001	0.22(0.09–0.53)	0.001
**Religion**				
Other	1.00		1.00	
Christian	0.15(0.05–0.42)	< 0.001	0.51(0.01–0.36)	0.003
Muslim	0.26(0.08–0.84)	0.025	0.07(0.01–0.55)	0.012
**Sexual partners**				
No	1.00		1.00	
Yes	4.76(3.11–7.28)	< 0.001	4.48(2.60–7.75)	< 0.001
**Attitude regarding FP services**				
Good	1.00		1.00	
Poor	0.28(0.18–0.43)	< 0.001	0.39(0.24–0.64)	< 0.001
**Model 2– Males**				
**Live with parents**				
No	1.00		1.00	
Yes	0.23(0.07–0.80)	0.020	0.19(0.05–0.67)	0.010
**Overall knowledge**				
Poor	1.00		1.00	
Good	1.62(0.74–3.51)	0.221	2.28(1.02–5.32)	0.050

OR: odds ratio, CI: confidence interval

### Qualitative findings

#### Characteristics of participants

A total of 5 in-depth interviews and 4 FGDs were conducted with a total of 30 young people; 10 were university students, whereas 20 were from the community. The focus groups were homogeneous in nature, for males and females separated. Two focus groups of 10 participants for males and females were conducted in the community, and two groups of five males and females were conducted from the University youths. The participants were young people aged 15 to 24 years. Themes were obtained through finding similar texts, patterns, and insights. We generated 8 different codes, 7 subthemes, and 3 themes.

#### Theme 1: knowledge of family planning methods

Majority of the young people did not have adequate knowledge regarding access and use of FP services. This was evidenced as most participants from the community reported that many young people used off label benefits of paracetamol and traditional herbal medicines for contraception. Additionally, many reported their source of information to be friends who seemed not to have adequate knowledge as well. Here are some of the verbatim comments to support the results:

*“After having sex today, you can take 4 Panadol tablets immediately after having sex or 6 tablets, though it depends, you can also take it a day after having the sexual intercourse, taking on the third day will be late for it to work well in preventing the pregnancy”. (Female, 22 years, Lira Town, Feb 2023)*

*“When I was in Primary six class. I was living with my sister and she had maids who told me about Panadol use”. (Female, 21 years, Junior quarters, Feb 2023)*

*“Some girls use paw paw leaves, others mixing diclofenac drug with herbs which can also cause abortion. But these procedures can also either lead to incomplete abortion, death or even over bleeding” (Male, 24 years, Barapwo, Feb 2023)*.

*“There is no proper sexual information. In the past, parents called children to prepare for education but today nowhere it’s practiced. Now it is only in schools to ensure that people know that sex is good but has challenges” (Female, 23 years, LU, Feb 2023)*.

#### Theme 2: beliefs about youth contraception

The majority of the participants also reported negative perceptions regarding family planning services. However, this appears to stem from the common narrative that frames sexual health for young people as taboo. To continue, many young people and the community reported distancing themselves from reproductive health programs, citing that their motives are not entirely transparent. Here are some of the quotes that were recorded to emphasize the narrative:

*“I see no meaning in engaging in such because they are just avenues for disseminating homosexuality and encouraging the youths to abort. They come in the sense of advocating for rights but instead teach that abortion and homosexuality is okay and a human right” (Female, 24 years, LU, Feb 2023)*.

*“Family planning services are for big people. But there is need for a comprehensive guidance in matters of Sexual health for adolescents and adults about hygiene and opposite sex interaction” (Male, 16 years, Lira town, Feb 2023)*.

#### Theme 3: friendliness of family planning services

Most of the participants reported that reproductive health services for young people are not friendly. The services are provided in environments that do not guarantee privacy and confidentiality, as well as during inflexible hours. To emphasize the narrative, here are a few verbatim comments:

It’s very difficult to go and access family planning services like pills from the teaching hospital…, can you imagine being served by your own lecturer who discourages having sex before marriage……Hmmm it’s funny! (Female, 24 years, LU, Feb 2023)

A friend can help buy contraceptives if the user is known to the health worker who is selling. The seller might inform the buyer’s parents when one goes to buy condoms. (Male, 18 years, Amuca, Feb 2023)

## Discussion

The study aimed to assess perceived access to FP services and associated factors among young people in Lira City, Northern Uganda. Though many models have been suggested to measure access, they have all showed deficiencies in measuring actual access to family planning methods [30]. This study adopted the Penchansky and Thomas (1981) framework that measures perception of access through a 5-item index to explore the level of perceived access in this study. Findings of the current study showed that good perceived access to FP services was among 31.7% of respondents, with 64.6% reporting availability, 76.5% accessibility, 61.3% acceptability, 66.7% accommodative and 87.9% affordability of FP methods at health facilities. Our study indicates a low perceived access to FP services. Among the various components, availability, acceptability and accommodation pose significant obstacles to contraceptive access. A similar study in South Africa also reported the accommodation component as the greatest obstacle for accessing FP services due to integrated care, long waiting hours, and limited operational hours [38]. Additionally, the study reported that community were less concerned about the availability of trained service providers and a variety of contraceptive methods [38]. These possibly explain the low perceived access in the current study. In line with the current study, a recent study on utilization of sexual and reproductive services including family planning among young people in Lira city also reported a low level of 42% [39].

The overall perceived access to FP services at 31.7% suggests a substantial gap in service availability, indicating the need for targeted interventions to enhance accessibility. The presence of different FP methods at health facilities (64.6%) is a positive aspect, but the study unveils underlying challenges that contribute to the overall low perceived access. One of the key positive findings is the proximity of health facilities for 79.3% of participants, emphasizing the importance of physical accessibility. Additionally, positive perceptions towards use of family planning commodities, such as acceptability of FP use by the young people (61.3%) and a conducive environment at health facilities (66.7%), indicate a foundation upon which interventions can build. However, challenges identified, particularly for females, including a lack of privacy (57.7%), fear of mistreatment by staff (77.2%), and difficulties in decision-making regarding FP use (66.2%), highlight the nature of barriers to access. These challenges align with existing literature on the importance of privacy [40], quality of service [41], and decision-making autonomy in shaping individuals’ willingness to utilize FP services [42].

Quantitative findings revealed significant associations between perceived access to FP services and various sociodemographic factors, emphasizing the complexity of the issue. For females, urban residence, religion, having sexual partners, and perception were identified as influencing factors, while for males, living with parents and overall knowledge played a significant role. These associations underline the necessity for tailored interventions that consider the specific challenges faced by each gender. Qualitative findings highlighted insufficient knowledge, negative perceptions, and unfriendly FP services. These findings provide a deeper understanding of the barriers, emphasizing inadequate knowledge of FP methods, negative cultural and societal perceptions about youth contraception, and unfriendly service environments. These findings are consistent with existing literature, highlighting the role of cultural perceptions, knowledge gaps, and service quality in shaping young people’s access to FP services [43]. In agreement with previous studies, the study underscores the importance of comprehensive sexual education programs and youth-friendly service initiatives [44]. Our study shows a notable link between Islam and Catholicism and perceived access to FP services, aligning with previous research on religious influences that notes that the use of contraception is not promoted by any of the two religions [45]. Further exploration and comparative analysis with other studies may help elucidate these discrepancies and provide a more nuanced understanding of the factors influencing access to FP services among young people in Northern Uganda.

### Strength and limitations

The study benefits from a mixed-methods approach, which integrates both qualitative and quantitative data to offer a comprehensive understanding of the factors influencing young people’s perceived access to family planning services. However, the cross-sectional design presents a limitation as it hinders the establishment of causality, providing only a snapshot of the situation at a specific moment and limiting exploration of temporal relationships over time. Acknowledging the small sample size and the potential bias introduced by selecting individuals with extensive knowledge on the topic, we recognize the limitation on the generalizability of our findings only to Lira City. The selection of individuals for IDIs may have inadvertently limited the diversity of perspectives represented in our study. Furthermore, participants may exhibit social desirability bias, particularly in studies addressing sensitive topics like sexual and reproductive health. Recall bias among participants, particularly when recalling past experiences related to sensitive topics or events that occurred some time ago, is also a possibility. Lastly, the quantitative sample was skewed towards females and those aged 15–19 years, potentially affecting the representativeness of the findings.

## Conclusion

Our study reveals a substantial gap in perceived access to family planning services among young people. Despite high awareness, barriers like privacy concerns and fear of mistreatment contribute to low access. Tailored interventions are needed, focusing on urban service access, religious beliefs for females, and knowledge enhancement for males. Positive aspects, such as diverse FP methods and physical accessibility, form a foundation for interventions. The study emphasizes the importance of youth-friendly services, comprehensive sexual education, and further research for a nuanced understanding and targeted interventions in Northern Uganda.

### Electronic supplementary material

Below is the link to the electronic supplementary material.

Supplementary Material 1

Supplementary Material 2

## Data Availability

The data for the study is not publicly available due to restrictions from the Research Ethics Committee (REC) for posting of public data. However, can be accessed from the principal investigator on a reasonable request (ekigongo@lirauni.ac.ug).

## Abbreviations

SRHR: Sexual Reproductive Health and Rights
SSA: Sub-Saharan Africa
VHT: Village Health Team
WHO: World Health Organization
