# Supplementary material for: Access to family planning services and associated factors among young people in Lira city northern Uganda
Source: BMC Public Health. 2024 Apr 24;24:1146. doi: 10.1186/s12889-024-18605-8 (PMC11044454; doi:10.1186/s12889-024-18605-8)
Supplement: Supplementary file 1 — Supplementary Material 1 [file 12889_2024_18605_MOESM1_ESM.docx]

**Questionnaire for “Access to Family Planning Services and associated factors among young people in Lira city”**

**Inclusion Criteria**

1. Male or female aged 15 to 24 years
2. Having lived in Lira city for the past six months
3. Present at the selected household
4. Provide consent of participation

**Questionnaire [Instructions:** Select the option that applies for each of the questions.]

|  | **Questionnaire number** | **……………………………………** |
| --- | --- | --- |
|  | **SECTION A: Socio demographic variables** |  |
| A1 | What is your Sex? | 1. Male 2. Female |
| A2 | What is your age in completed years? | ……………………. |
| A3 | What is your level of education? | 1. Primary 2. Secondary 3. Tertiary 4. None |
| A4 | What is your mother’s education level? | 1. Primary 2. Secondary 3. Tertiary 4. None |
| A5 | What is your father’s education level? | 1. Primary 2. Secondary 3. Tertiary 4. None |
| A6 | What is your place of residence? | 1. Rural 2. Urban |
| A7 | What is your religious affiliation? | 1. Anglican 2. Catholic 3. Muslims 4. Others |
| A8 | What is your marital status? | 1. Married 2. Single 3. Separated 4. Cohabiting |
| A9 | Are you living with your parents? | 1. No 2. Yes |
| A10 | What is your employment status? | 1. Employed 2. Self employed 3. Un employed 4. Student |
| A11 | What is your estimated monthly income in UGX? | ………………………… |
| A12 | Do you have a child? | 1. No 2. Yes |
| A13 | What is your father’s occupation? | 1. Peasant farmer 2. Teacher 3. Business man 4. Health worker 5. Engineer 6. Politician 7. Others specify …… |
| A14 | What is your mother’s occupation? | 1. Peasant farmer 2. Teacher 3. Business man 4. Health worker 5. Engineer 6. Politician 7. Others specify ……… |
|  | **SECTION B: Sexual behavior** |  |
| B1 | Have you ever had sexual intercourse? | 1. No 2. Yes |
| B2 | If yes, when did last have a sexual encounter | ……………… |
| B3 | Have you ever been forced into sexual activity or raped any of your partners at any time? | 1. No 2. Yes |
| B4 | How many sexual partners do you have currently? | …………………………… |
| B5 | Are you currently using any contraceptive method? | 1. No 2. Yes |
|  | **SECTION C: Utilization of FP services** |  |
| C1 | Are the family planning commodities available when you need them, and meet your FP needs? | 1. No 2. Yes |
| C2 | Is the location of the facilities that provide family planning services convenient for you? | 1. No 2. Yes |
| C3 | Are the characteristics of the family planning providers (including attitudes and attributes such as age, sex and religion) comfortable for you? | 1. No 2. Yes |
| C4 | Do health providers organize FP services in ways (inclusing appointment system, hours of operation and environment) that suit your preferences? | 1. No 2. Yes |
| C5 | Do you have to pay for family planning services? | 1. No 2. Yes |
|  | **SECTION D: Knowledge regarding FP services** |  |
| D1 | Are you aware of availability of family planning services? | 1. No 2. Yes |
| D2 | Do you know at least three family planning methods?  [Hint: Ask participant to mention them] | 1. No 2. Yes |
| D3 | Do you know the health facilities that offer family planning services?  [Hint: Ask participant to mention some of them in the residence] | 1. No 2. Yes |
| D4 | Do you know the health facilities that offer abortion services?  [Hint: as participant to mention some of them in the residence] | 1. No 2. Yes |
| D5 | Do you know the health facilities that offer STIs services?  [Hint: ask participant to mention some of them in the residence] | 1. No 2. Yes |
| D6 | Do you know how to prevent pregnancy? | 1. No 2. Yes |
| D7 | Do you know the rights of young people?  [Hint: Ask participant to mention some of them] | 1. No 2. Yes |
| D8 | Do you know the sexual and reproductive health rights of young people?  [Hint: Ask participant to mention some of them] | 1. No 2. Yes |
|  | **SECTION E: Perceptions regarding family planning services** |  |
| E1 | Family planning services are supposed to be used by only married people? | 1. No 2. Yes |
| E2 | Information at the health facility is kept confidential | 1. No 2. Yes |
| E3 | I can choose my own partner | 1. No 2. Yes |
| E4 | I can decide on using family planning method? | 1. No 2. Yes |
| E5 | I can access family planning services whenever I want | 1. No 2. Yes |
| E6 | There is not enough privacy at the health facilities | 1. No 2. Yes |
| E6 | I fear being embarrased at the health facilitiy | 1. No 2. Yes |
| E7 | I fear being mistreated by the staff at the health facility | 1. No 2. Yes |
