# Supplementary material for: Access to family planning services and associated factors among young people in Lira city northern Uganda
Source: BMC Public Health. 2024 Apr 24;24:1146. doi: 10.1186/s12889-024-18605-8 (PMC11044454; doi:10.1186/s12889-024-18605-8)
Supplement: Supplementary file 2 — Supplementary Material 2 [file 12889_2024_18605_MOESM2_ESM.docx]

**Qualitative guides for “Access to Family Planning Services and associated factors among young people in Lira city”**

**In-depth Interview Guide**

**Introduction:**

1. Welcome and introduce yourself.
2. Explain the purpose of the study and reassure confidentiality.
3. Ask for consent to proceed with the interview.

**Demographic Information:**

1. Can you tell me a bit about yourself? (age, gender, occupation, educational background)
2. Are you currently in a relationship?

**Knowledge and Awareness:**

1. What do you understand by the term "family planning"?
2. How familiar are you with the family planning services available in Lira city?
3. Have you ever used any family planning method? If yes, which one(s)?

**Attitudes and Perceptions:**

1. What are your thoughts or beliefs about using family planning methods?
2. What are some common myths or misconceptions you've heard about family planning?
3. How do you think family planning services could be improved in Lira city to better meet the needs of young people?

**Access and Utilization:**

1. Have you ever accessed family planning services in Lira city? If yes, what was your experience like?
2. If no, what factors have prevented you from accessing these services?
3. What do you think could encourage more young people to use family planning services?

**Closing:**

1. Is there anything else you would like to add or share about family planning services in Lira city?
2. Thank the participant for their time and participation.

**Focus Group Discussion Guide:**

**Introduction:**

1. Welcome participants and introduce yourself.
2. Briefly explain the purpose of the focus group and the rules (respect, confidentiality, etc.).
3. Icebreaker question: What comes to mind when you hear the term "family planning"?

**Knowledge and Awareness:**

1. What do you know about family planning services available in Lira city?
2. Have you or anyone you know ever used family planning services? What was their experience?

**Attitudes and Perceptions:**

1. How do you feel about the idea of using family planning methods?
2. Are there any cultural or religious beliefs in Lira city that influence people's attitudes towards family planning?

**Barriers and Facilitators:**

1. What do you think are the main barriers to accessing family planning services for young people in Lira city?
2. How can these barriers be overcome?
3. What would encourage more young people to utilize family planning services?

**Personal Experiences and Stories:**

1. Can anyone share a personal experience or story related to family planning?
2. How do you think family planning has impacted families or communities in Lira city?

**Closing:**

1. Any final thoughts or comments on family planning services in Lira city?
2. Thank participants for their time and contributions.
